# Immune response modulation by curcumin in a latex allergy model

**DOI:** 10.1186/1476-7961-5-1

**Published:** 2007-01-25

**Authors:** Viswanath P Kurup, Christy S Barrios, Raghavan Raju, Bryon D Johnson, Michael B Levy, Jordan N Fink

**Affiliations:** 1 Department of Pediatrics, Medical College of Wisconsin, 8701 Watertown Plank Road, Milwaukee, WI 53226, USA; 2 Department of Medicine, Medical College of Wisconsin, 8701 Watertown Plank Road, Milwaukee, WI 53226, USA; 3 Research Service, V A Medical Center, 5000 West National Avenue, Milwaukee, WI 53295, USA; 4 Neuromuscular Diseases Section, National Institute of Neurological Disorders and Stroke, National Institute of Health, Bethesda, MD 20892, USA; 5 University of Alabama School of Medicine, Department of Surgery and Department of Microbiology and Immunology, Volker Hall, Room G094, 1670 University Boulevard, AL 35294, USA

## Abstract

**Background:**

There has been a worldwide increase in allergy and asthma over the last few decades, particularly in industrially developed nations. This resulted in a renewed interest to understand the pathogenesis of allergy in recent years. The progress made in the pathogenesis of allergic disease has led to the exploration of novel alternative therapies, which include herbal medicines as well. Curcumin, present in turmeric, a frequently used spice in Asia has been shown to have anti-allergic and inflammatory potential.

**Methods:**

We used a murine model of latex allergy to investigate the role of curcumin as an immunomodulator. BALB/c mice were exposed to latex allergens and developed latex allergy with a Th2 type of immune response. These animals were treated with curcumin and the immunological and inflammatory responses were evaluated.

**Results:**

Animals exposed to latex showed enhanced serum IgE, latex specific IgG_1_, IL-4, IL-5, IL-13, eosinophils and inflammation in the lungs. Intragastric treatment of latex-sensitized mice with curcumin demonstrated a diminished Th2 response with a concurrent reduction in lung inflammation. Eosinophilia in curcumin-treated mice was markedly reduced, co-stimulatory molecule expression (CD80, CD86, and OX40L) on antigen-presenting cells was decreased, and expression of MMP-9, OAT, and TSLP genes was also attenuated.

**Conclusion:**

These results suggest that curcumin has potential therapeutic value for controlling allergic responses resulting from exposure to allergens.

## Background

Recent years have witnessed an increased prevalence of allergy and asthma among people in developed countries [1-4]. Although not of the same magnitude, similar increases in allergic diseases have also been observed in developing countries [5]. Search for novel treatments have significantly advanced in recent years. This increased attention has led to the exploration of alternative medicines with particular interest in plant products that have been in use for many years in the old world countries. Reviews published in recent years suggest that some of these folklore medicines have significant effect in reducing the severity of respiratory disease symptoms and improving patient's quality of life [6,7].

Alternative medicines, particularly plant extracts have shown acceptance by patients and physicians alike [6-8]. However, no detailed scientific studies have been conducted to further the understanding of the anti-allergic mechanisms associated with these products. In spite of the lack of information, a substantial interest has been shown to alternative and supplementary medicines. Currently, closer to 2,000 herbal products are available for the treatment of various ailments and the list is steadily growing [6,7].

A number of herbs and herbal products have been used in the treatment of allergy and asthma in ancient traditional Chinese medicine, Indian Ayurvedic medicine, and Japanese Kampo medicine [8-14]. However, few scientific studies have been carried out to ascertain their action and effectiveness [15,16]. In recent years, turmeric, a spice used in Asian countries, has attracted the attention of researchers due to its reported effectiveness in inflammatory and other disorders [17,18]. The effectiveness of curcumin, the active component of turmeric, has been evaluated in various diseases, but not for asthma and allergy, in spite of the fact that it has been used in the treatment of asthma and allergy for many centuries [17-19]. Here, we report our findings on the immunomodulatory role of curcumin in a mouse model of latex allergy. The results indicate that curcumin downregulated Th2 responses and reduced lung inflammation in latex sensitized mice, suggesting a possible role for curcumin in controlling allergic responses.

## Materials and methods

### Sensitization of Mice with Latex Allergens

Latex allergy in BALB/c mice was induced according to a protocol described previously [20-22]. In brief, 8–10 week old BALB/c mice were divided into three groups. The first group (Group 1) was challenged with latex allergens, the second group (Group 2) was challenged with latex and treated with curcumin (Sigma Chemicals), and the third group (Group 3) consisted of controls treated with curcumin only. 100 μg of a Malaysian non-ammoniated (MNA) latex extract, isolated from sap collected from the rubber plant *Hevea brasiliensis*, was injected intraperitoneally into the mice, once a week for two weeks. Remaining challenges were done intranasally twice a week for four weeks (50 μg of latex in 30 μl of PBS per challenge) (Groups 1 and 2). Intranasal inoculations with latex antigen and intragastric administration of curcumin (250 μg in 250 μl PBS) (Group 2) were carried out after anesthetizing the mice with xylazine. Control animals (Group 3) received PBS intranasally and curcumin intragastrically. The levels of total serum IgE and latex specific IgG_1 _were measured by ELISA as described previously [22]. When a significant antibody response was detected, the animals were challenged with a final dose of latex allergens, and euthanized 48 hours later. Blood, lung tissue, and spleens were collected and evaluated as described below. The animal studies were approved by the Veterans Affairs Animal Care Committee.

### Total Serum IgE

Total serum IgE levels were determined in all mice before sensitization and after euthanization as previously reported [22,23]. Serum IgE levels was expressed as ng/ml after comparing the optical density (O.D. at 480_nm_) values to mouse IgE standards.

### Latex Specific IgG_1 _and IgG_2a _in the Sera of Mice

Levels of latex antigen-specific IgG_1 _and IgG_2a _in collected serum samples were studied by ELISA as previously described [22]. In brief, micro titer plate wells (Immulon II, Fisher Scientific, Itasca, IL) were coated with 5 μg/ml of latex proteins in PBS (pH 7.4). The plates were incubated at room temperature for 3 hours, followed by overnight incubation at 4°C. The plates were then washed with PBS and after blocking the wells with 0.5% Bovine serum albumin in PBS, 100 μl of serum diluted in PBS containing 0.05% Tween 20 (PBS-T) was added to the wells, and the plates incubated at room temperature for three hours. The wells were washed with PBS-T and isotype specific biotinylated anti-mouse antibody was added for an additional hour. The plates were washed again and streptavidin conjugated horse radish peroxidase was added to the wells for one hour. After washing the plates, the substrate was added and the color developed with O-phenylene diamine (OPD). The color development was stopped by adding 2N H_2_S04, and the optical density (O.D.) read at 490_nm _using an ELISA reader. The O.D. values of several serum dilutions were used to calculate log_10 _titer, and the different groups were compared.

### Eosinophils

Peripheral blood eosinophils were plated on slides stained with Eosin-Y and enumerated using a hemacytometer [23]. Eosinophil numbers were assessed before and during sensitization and at the end of the experiment.

### Lung Histology

Immediately after sacrifice, the lungs were inflated with 10% neutral buffered formalin to prevent atelectasis. The specimens then were fixed in formalin and processed. Sections were cut at 5 μm thickness and stained with hematoxylin-eosin and PAS. Lung inflammation was scored with special reference to the infiltrating cell types and the severity of lesions as described previously [22-24].

### Immunohistochemistry

Lung sections were examined for cells producing IFN-γ, IL-4, IL-5, IL-10, and IL-13 by immunohistochemistry as previously described [25]. Lungs were frozen in liquid nitrogen and frozen sections from different groups of mice were fixed in 4% paraformaldehyde. The sections were incubated in 0.3% H_2_O_2 _in PBS for 15 minutes. After washing three times with PBS for five minutes each, the sections were blocked with PBS containing 5% bovine serum albumin (BSA) for three hours. The sections were then incubated for two hours at room temperature with 1:20 diluted biotinylated anticytokine antibodies (Pierce, R&D) in PBS containing 3% BSA. The slides were then incubated for 30 minutes at room temperature with streptavidin peroxidase (1:50 diluted). The color was developed with 3,3-diaminobenzidine tetra chloride (DAB) (Sigma). Numbers of cytokine positive cells were determined by counting five different microscopic fields.

### Spleen Cells

Spleens were processed into single cell suspensions and antigen dependent proliferation studied by tritiated thymidine uptake [26]. Briefly, spleen cells (1 × 10^5^/well) were cultured for seven days in 96 well plates in 200 μl of RPMI 1640 medium supplemented with glutamine, sodium pyruvate, 10% heat inactivated fetal bovine serum (FBS), and penicillin and streptomycin (complete RPMI). Latex antigen (5 μg/ml) or Concanavalin A (5 μg/ml) was added to experimental wells [27]. One μCi of ^3 ^[H] thymidine was added for the final 18 hours of culture. The incorporated ^3^H thymidine was measured by liquid scintillation counting, and the stimulation indices (SI) were calculated as described before [26].

### Cytokine Production by Spleen Cells

Spleen cells (1 × 10^7^) were placed in complete RPMI and cultured in 24 well plates for 60 hours. Latex antigen (5 μg/ml) was added to experimental wells at the beginning of culture. After incubation, cell free supernatants were collected and analyzed for cytokines by ELISA, including IL-4, IL-5, IL-10, IL-13, and IFN-γ [26].

### Flow Cytometric Analysis of Lung Cells

Lungs were removed aseptically and cut into small pieces of about 2 to 3 mm in size. The pieces were digested enzymatically by treating with 120 μg/ml Dispase (Invitrogen) and Collagenase (Sigma) for one hour at 37°C. After incubation, the tissue was homogenized gently in a tissue grinder and the cells were collected. These cells were washed three times, and the lung cells for each group were pooled (equal numbers from each mouse) and suspended in complete RPMI. The cells were stained with combinations of FITC and PE conjugated antibodies specific for CD4, CD8, CD25, CD28, CD80, CD86, CD152, OX40, B220, or Mac-1 (26). The stained cells were run through a FACS Calibur flow cytometer (Becton-Dickinson, Mountain View, CA) and analyzed using FlowJo software (Tree Star, San Carlos, CA).

### RNA Isolation

Total RNA was isolated using RNeasy mini kits (Qiagen, Valencia, CA) [28]. Briefly, lung tissues were homogenized using disposable pestles and tubes (Kontes Glass Company, Vineland, NJ) in the presence of lysis buffer. Lysates were transferred to QIAshredder spin columns (Qiagen), spun for 2 minutes, the eluates collected, and RNA isolated as per the manufacturer's protocol. The RNA was quantified by measuring OD (Nanodrop ND-1000, Wilmington, DE).

### One Step RT-PCR

One-step RT-PCR reactions were performed in triplicate using TaqMan one-step RT PCR (Applied Biosystems, Branchburg, NJ) [29]. Briefly, the sequence specific FAM-labeled Taqman primer-probe pairs (Applied Biosystems, Foster City, CA) and 10 ng total RNA were mixed with reaction buffer supplied by the manufacturer in a 20 μl reaction volume. The sequential one-tube reverse transcription and real time PCR were performed in an Opticon 2 thermal cycler (MJ Research/Bio Rad Laboratories, Hercules, CA). The temperature conditions included an initial 48°C incubation for 30 minutes, followed by AmpliTaq Gold activation at 95°C for 10 min, 40 cycles of amplification at 95°C for 30 sec and 60°C for 1 min cycles. Glyceraldehyde-3-phosphate dehydrogenase (GAPDH; assay id: Mm99999915_g1) was used as an internal control. Applied Biosystem Taqman gene expression primer-probe pairs specific to lymphocyte antigen 75 (Ly5, Assay id: Mm00522144_m1), matrix metallopeptidase 9 (MMP9; Assay id: Mm00600163_m1), ornithine amino transferace (OAT; Assay id: Mm00497544_m1) and thymic stromal lymphopoietin (TSLP; Assay id: Mm00498739_m1) were used.

### Statistical Analysis

Total serum IgE levels, latex specific IgG_1 _and IgG_2a_, peripheral blood eosinophils, cytokine production and stimulation of spleen cells in response to latex antigens *in vitro *were compared among different groups of mice. The data were analyzed and compared using student 't' test with unequal variance and the results expressed as means ± SEM. 'P values' < 0.05 were considered significant. The flow cytometric studies were conducted with pooled lung cells and the results are presented as such. PCR values were calculated as the mean results of three separate mice.

## Results

### Total Serum IgE and Latex Specific IgG

**Figure 1:**
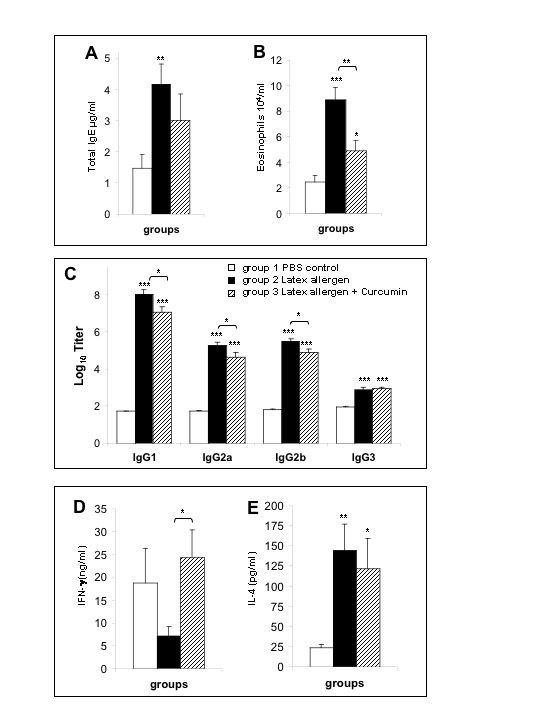
Total serum IgE, peripheral blood eosinophils, latex specific antibodies, and cytokine responses. A. Total serum IgE in nanogram per ml in controls (Group1); latex sensitized mice (Group 2); and latex sensitized mice treated with curcumin (Group 3). B. Peripheral blood eosinophils in the three groups. C. Serum IgG_1,2a,2b, _and IgG_3 _latex specific antibodies. The antibody levels were calculated from the O.D. values of at least five two-fold dilutions and log titers calculated using a computerized program. D. IFN-γ production (ng/ml) by antigen-stimulated spleens cells as measured by ELISA. E. IL-4 production (Pg/ml) by antigen-stimulated spleen as measured by ELISA.

There was a significant increase in total serum IgE levels in animals exposed to latex antigens as compared to controls (Fig. 1A). This increase was arrested in mice challenged with latex and treated with curcumin. However, the difference between curcumin treated and untreated mice was not statistically significant.

Latex specific IgG_1_, IgG_2a_, IgG_2b_, and IgG_3 _was readily detected in the serum of latex-sensitized mice (Fig. 1C). This increase in specific antibody was several fold greater than the baseline antibody levels detected in unexposed controls. The control mice treated with curcumin only (no latex sensitization) showed only baseline values.

### Peripheral Blood Eosinophils

The numbers of eosinophils in the peripheral blood of latex-sensitized mice were markedly elevated as compared to PBS controls (Fig. 1B). Notably, curcumin treatment significantly decreased the numbers of eosinophils in latex sensitized mice (p < 0.05). Control mice treated with curcumin alone had normal numbers of eosinophils.

### Antigen Induced Lymphocyte Stimulation

Latex allergens (MNA) were unable to induce the proliferation of spleen cells from latex sensitized mice (data not shown), possibly due to toxicity of the latex extract preparation [19]. In contrast, the recombinant latex allergen, Hev b 6 was used to stimulate spleen cells, enhanced proliferation was detected in cells from latex antigen exposed mice (data not shown). Curcumin treatment of latex sensitized mice only marginally affected Hev b 6 induced lymphocyte proliferation (data not shown). Concanavalin-A induced stimulation of lymphocytes from latex challenged mice showed reduced proliferation as compared to controls, and this decreased proliferation was partially restored in latex challenged mice treated with curcumin (results not shown).

### Cytokine Production by Spleen Cells

Cytokines were not detected in the culture supernatants of cultured spleen cells from curcumin treated or PBS treated control mice (Fig. 1D &1E). Spleen cells stimulated with Hev b 6 also failed to produce detectable levels of cytokines. Varying levels of cytokines were detected in the culture supernatants of antigen stimulated cells and from latex sensitized mice treated with curcumin. Reduced levels of IFN-γ were detected from cells of latex sensitized mice compared to cells from normal mice, while increased amounts of IFN-γ were produced from the cells of mice challenged with latex and treated with curcumin (Fig. 1D). Although, IL-4 production was only slightly reduced in culture supernatants from latex sensitized mice treated with curcumin (Fig. 1E), overall the cytokine profiles indicated that curcumin shifted the latex-induced Th2 response towards a Th1 type of response. No major differences were detected for IL-5, IL-10, and IL-13 (results not shown).

### Analysis of Lung Tissue

**Figure 2:**
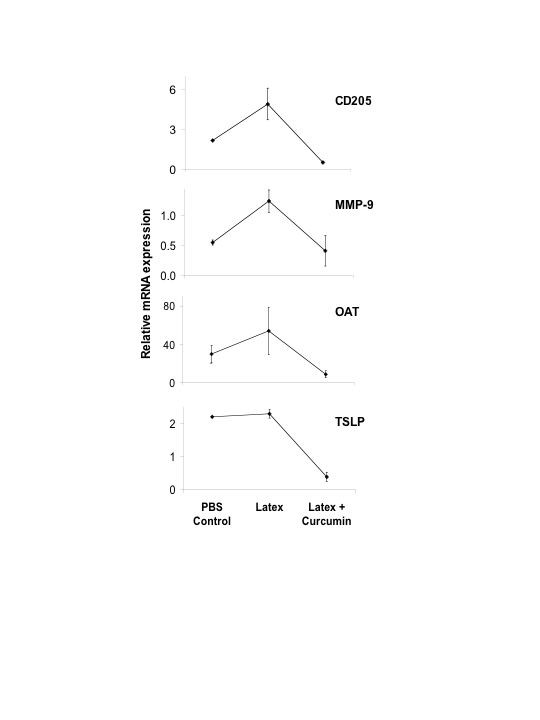
Relative mRNA expression of CD205, MMP-9, OAT, and TSLP in the lungs of control and experimental mice.

Messenger RNA was isolated from the lung tissue and studied for Ly75 (CD205), MMP9, OAT and TSLP expression. The results indicated that there were diverse responses in the different groups of mice (Fig. 2). Expression of OAT, MMP9, and Ly75 (CD205) were increased in latex sensitized mice as compared to untreated PBS controls, while TSLP levels were similar. There was a marked reduction in the expression of all four genes in latex sensitized mice treated with curcumin.

### Flow Cytometric Analysis of Lung Cells

**Figure 3:**
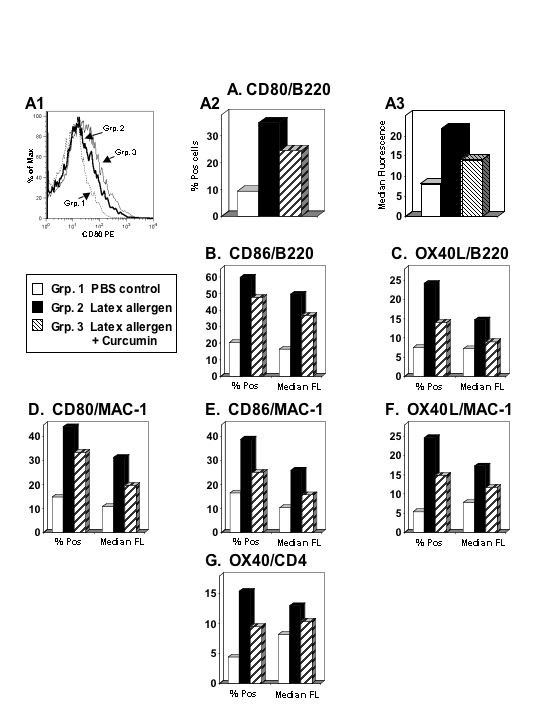
Expression of costimulatory molecules on cells from the lungs of control, latex-sensitized, and latex-sensitized mice treated with curcumin. Lung cells were pooled from mice in each group and analyzed by flow cytometry. Representative histograms comparing CD80 expression on B cells from the three groups tested are shown in panel A1. CD80 expression is shown as percent positive (% Pos) cells in Panel A2, and as median fluorescence (FL) values in Panel A3. The percentages of positive cells and median fluorescence values are shown for CD86 and OX40L on B cells in Panels B and C, respectively. Similarly, CD80, CD86, and OX40L expression on lung macrophages is shown for the three groups in Panels D, E, and F. Percentages of CD4^+ ^T cells expressing OX40 and the median fluorescence values for OX40 are shown in Panel G.

Expression of costimulatory molecules on lung cells from experimental mice were examined by flow cytometry, including CD28, OX40, and CTLA-4 on T-cells, and CD80, CD86, and OX40L on B cells and macrophages. A representative histogram shown in panel A1 of Figure 3 depicts CD80 expression on lung B cells from the different treatment groups. Increased percentages of B cells expressing CD80 were detected in the lungs of latex-sensitized mice as compared to PBS controls (Fig. 3A2). Curcumin treatment reduced the expression of CD80 on lung B cells of latex-sensitized mice (Fig. 3A1), and the reduced expression was reflected by decreased percentages of lung B cells expressing CD80 (Fig. 3A2) and decreased CD80 median fluorescence values (Fig. 3A3). Lung B cells from mice sensitized with latex and treated with curcumin also showed reduced CD86 and OX40L expression as compared to lung B cells from latex-sensitized mice (Fig. 3B and 3C, respectively), and the expression of CD80, CD86, OX40L on lung macrophages exhibited a similar pattern (Fig. 3D–F). Finally, percentages of lung CD4^+ ^T cells expressing OX40 and OX40 median fluorescence values were decreased in latex-sensitized/curcumin treated mice as compared to latex-sensitized mice (Fig. 3G). Curcumin treatment also resulted in slightly reduced percentages of lung CD4^+ ^T cells expressing CTLA-4 (21.6% vs. 29.3% in latex-sensitized mice as compared to 7.6% in control mice), while no differences were observed in regard to CD28 expression (data not shown).

### Lung Histology

**Figure 4:**
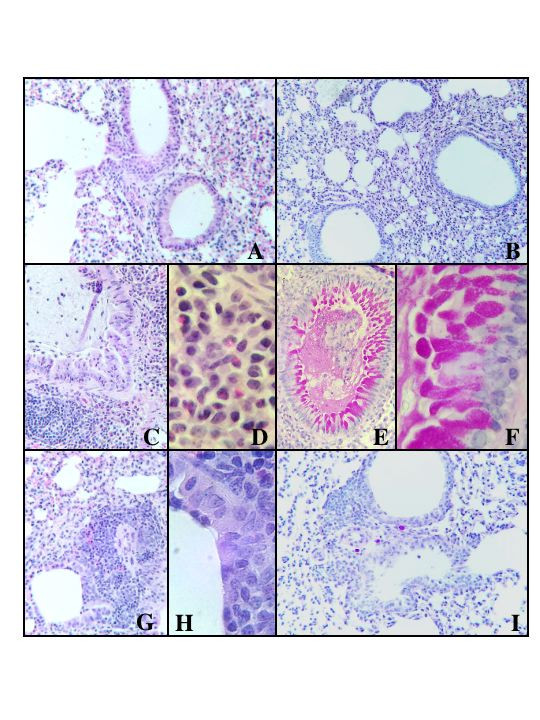
Histology of the lungs studied from control and experimental mice. A. Lungs of the control mice stained by Hematoxylin and eosin (H&E) ×40. B. Lung tissues stained with PAS ×40. C. Latex challenged mice, H&E at ×40. D. Latex challenged mice at ×400. E. Latex challenged mice stained with PAS at ×400. F. Latex challenged mice stained with PAS at ×400. G. Lung section from curcumin treated mice (Group 3), H&E at ×40. H. Lung sections from curcumin treated mice but magnification H&E at ×400. I. Lung section from curcumin treated mice stained with PAS at ×40.

Mice sensitized with latex antigen showed significant interstitial inflammation with peribronchiolar and perivascular infiltrates (Fig. 4). The inflammatory cells primarily consisted of small lymphocytes with plasma cells and epitheloid histiocytes. In PAS stained sections, increased numbers of bronchial epithelial cells, particularly PAS positive goblet cells were discernable (Fig. 4B, E, F &4I). Bronchial epithelial cell hyperplasia was predominant in latex challenged mice (Fig. 4C). A marked increase in eosinophils was evident in latex challenged mice (Fig. 4C &4D), but was considerably reduced in latex challenged mice treated with curcumin (Fig. 4G &4H). In the curcumin treated, latex sensitized mice there was only minimal perivascular edema and moderate perivascular cuffing with infiltration of neutrophils and mononuclear cells. These mice also had fewer lesions consistently devoid of eosinophils, although some neutrophils and mononuclear cells were discernible (Fig. 4G &4H). There was also less bronchial epithelial hyperplasia and no goblet cells (Fig. 4G, H &4I). Control mice treated with PBS and curcumin showed normal lung pathology (Fig. 4A &4B).

### Immunohistochemistry

**Figure 5:**
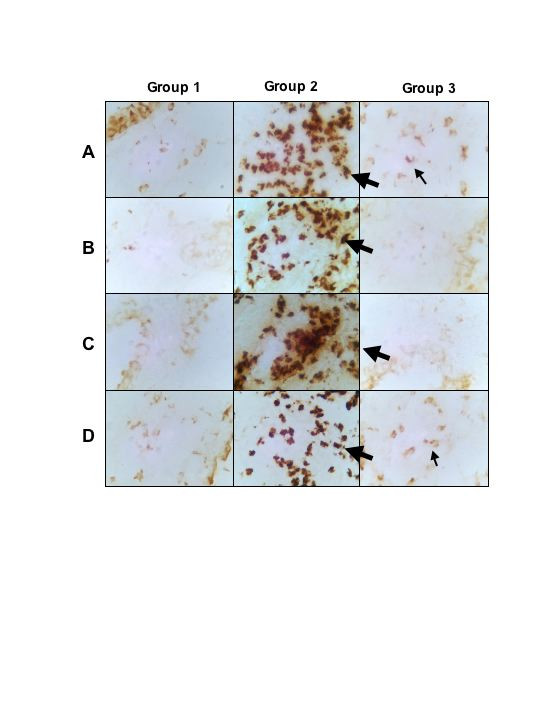
Immunohistochemical staining for IL-4, IL-5, IL-13, and IFN-γ of control mice (Group 1), latex challenged mice (Group 2), and latex challenged treated with curcumin (Group 3). A. IL-4; B. IL-5; C. IL-13; and D. IFN-γ.

Lung tissue sections were stained for IL-4, IL-5, IL-10, IL-13 and IFN-γ. Cytokine producing cells in the lungs of PBS-treated control mice could only be detected in low numbers for IL-10 and IFN-γ (Fig. 5D). There was a marked increase in lung cells secreting IL-4, IL-5, IL-13, and IFN-γ in the latex-challenged mice (Fig. 5A, B, C, D). Curcumin treatment of latex sensitized mice resulted in a marked decrease in IL-4, IL-5, and IL-13 expressing cells (Fig. 5A, B &5C).

## Discussion

Intranasal challenge with latex allergens induced a strong IgE and eosinophil response with characteristic inflammatory changes in the lungs of mice as reported previously [21,22]. Other features of this model included enhanced IL-4 secretion by antigen stimulated spleen cells and reduced production of IFN-γ. We also detected slightly enhanced mRNA levels of Ly75 (CD-205), MMP-9, and OAT in mice challenged with latex allergens. Immunohistochemistry consistently revealed increased numbers of cells secreting Th2 cytokines in the lungs of mice particularly, IL-4, IL-5, and IL-13. All these features are significant biomarkers detected in allergic subjects and particularly in latex allergy [30]. One of the more striking features was the accumulation of large numbers of inflammatory cells in the perivascular and peribronchiolar regions of the lung parenchyma. The infiltrating cells included lymphocytes, macrophages, and occasional neutrophils with strikingly large numbers of infiltrating eosinophils. Co-stimulatory molecules including CD80, CD86, and OX40L all had increased expression in latex sensitized mice as compared to normal mice [26]. Thus, the model has all the distinguishing features of IgE mediated allergy.

Mice exhibiting allergic responses to latex upon treatment with curcumin showed either reduced expression of several Th2 parameters or they remained unchanged from normal control mice. It is interesting that no major differences were noted in the antibody levels of the latex challenged mice from those sensitized with latex and treated with curcumin. However, total serum IgE was reduced in the latter group of mice. A complete disappearance of eosinophils in the lungs and a consistent reduction in the inflammatory response as indicated by fewer inflammatory loci were two of the more remarkable features in curcumin-treated, latex-sensitized mice. Reduced expression of co-stimulatory molecules on T-lymphocytes, B-lymphocytes, macrophages, and granulocytes was also noted.

Taken together, our data indicate that curcumin is capable of down regulating the allergic response in mice challenged with latex allergens. Although some of the Th2 responses were only marginally reduced in curcumin-treated, latex-sensitized mice, other Th2 parameters were strikingly reduced. The number of IFN-γ expressing cells in the lungs of latex treated mice also increased indicating an admixture of both Th1 and Th2 responses in this model. It is possible that the lung inflammation may be a result of the IFN-γ mediated Th1 response (Fig. 4C, D, E &4F), while the type 1hypersensitivity is the result of a Th2 cytokine and IgE response [24,31,32]. The fewer lesions and less inflammation in the lung of curcumin-treated, latex-sensitized mice support this contention. IFN-γ levels in the lungs of these animals were much lower than those detected in latex challenged and curcumin treated mice where IFN-γ secreting cells were almost comparable to normal control mice treated with curcumin only. This may have profound influence in eliciting the allergic inflammation, a switch from a Th2 cytokine profile in acute lesions to increased IFN-γ levels and high numbers of cytolytic T cells in chronic lesions, while type 2 cytokines still remain high. The results suggest that curcumin reduced the IFN-γ producing cells in the lung resulting in the reduced inflammation that was detected in latex-sensitized mice. Curcumin treatment also reduced various Th2 cytokines producing lung cells.

Ly75 (CD-205), MMP-9, and OAT were all at lower levels in latex-sensitized mice treated with curcumin compared to those not treated with curcumin. Curcumin treated mice also showed reduced expression of TSLP. The reduced TSLP may result in lower IFN-γ production, which then results in a reduced inflammatory lung response [24,33-35]. The reduced TSLP levels further substantiate the reduced inflammatory response induced by curcumin. Arginase metabolism results in the production of ornithine, which produce proline by the activity of the enzyme OAT. Proline inhibits collagen production in the lungs and, therefore, may be directly involved in airway remodeling [36]. Curcumin may have a direct effect on this process by down regulating OAT. In previous reports by us and others, arginase has been shown to be markedly increased in *Aspergillus *induced allergy [37,38]. Upregulation of enzymes in arginine metabolism may also imply an enhanced nitric oxide (NO) production[36]. MMP-9 correlates with enhanced tissue destruction in allergic airway disease [39], and MMP-9 was markedly increased in latex challenged mice, but showed a reduction in curcumin treated latex sensitized mice. Thus, the inflammatory markers of lung inflammation showed an overall reduction in latex sensitized and curcumin treated mice.

Previous studies have indicated that curcumin reduces inflammation through inhibition of STAT3 phosphorylation [40]. These same findings also indicated that curcumin does not inhibit STAT5 or IFN-γ inducible STAT1 expression. However, curcumin has been shown to inhibit *Dermatophagoides farineae *induced IL-4 and IL-5 production similar to what we observed in the present study [15]. It has been reported that NO production by *Leishmania *was decreased in curcumin treated BALB/c mice infected with *Leishmania *larvae. This reduction in NO is significant as it is a salient feature of asthma and allergy [41].

## Conclusion

The results presented in this report clearly suggest that curcumin, the active ingredient of turmeric, is capable of reducing or suppressing the Th2 response induced in mice by exposing them to latex allergens. Although the exact mechanism is not clearly understood, it is possible that a number of different mechanisms are concurrently at work. This may include suppression of Th2 responses as evidenced by reduced IL-4 and IL-13 production and depletion of eosinophils in the lungs, and attenuation of lung inflammation through expression of molecules such as TSLP, MMP-9, and OAT. The results indicate that curcumin has potential therapeutic value in allergy. However, further studies are needed to understand the exact mechanism involved in curcumin induced suppression of allergic responses.

## Abbreviations

PBS – phosphate buffered saline; OD – optical density; ELISA – enzyme linked immunosorbent assay; NO – nitric oxide; PAS – periodic acid Schiff; DAB – 3-3-Diaminobenzidine tetra chloride; MNA – Malaysian non-ammoniated latex; OAT – ornithine amino transferase; MMP – matrix metalloproteinase; TSLP – thymic stromal lymphopoitin

## Declaration of Conflicting interests

The author(s) declare that they have no competing interests.

## Authors' contributions

VPK was responsible for the overall planning of the experiments. CSB contributed towards animal experiments and cell culture studies. RR conducted the molecular biology studies. BDJ carried out the flowcytometric studies. MBL and JNF contributed in the planning and discussions. All the authors contributed to the manuscript preparation.
